# NLRP3 associated with chronic kidney disease progression after ischemia/reperfusion-induced acute kidney injury

**DOI:** 10.1038/s41420-021-00719-2

**Published:** 2021-10-29

**Authors:** Zhihuang Zheng, Kexin Xu, Chuanlei Li, Chenyang Qi, Yili Fang, Nan Zhu, Jinfang Bao, Zhonghua Zhao, Qing Yu, Huijuan Wu, Jun Liu

**Affiliations:** 1 Department of Nephrology, Shanghai General Hospital, Shanghai Jiaotong University School of Medicine, Shanghai, China; 2 Department of Pathology, School of Basic Medical Sciences, Fudan University, Shanghai, China; 3 Department of Nephrology, Shanghai Tenth People’s Hospital, Tongji University School of Medicine, Shanghai, China

**Keywords:** Prognostic markers, Acute kidney injury

## Abstract

Nod-like receptor protein 3 (NLRP3), as an inflammatory regulator, has been implicated in acute kidney injury (AKI). Failed recovery after AKI can lead to chronic kidney disease (CKD). However, the role of NLRP3 in the AKI-CKD transition is still unknown. A mild or severe AKI mouse model was performed by using ischemia-reperfusion injury (IRI). We evaluated the renal NLRP3 expression in acute and chronic phases of ischemic AKI, respectively. Although serum creatinine (Cr) and blood urea nitrogen (BUN) levels in AKI chronic phase were equivalent to normal baseline, histological analysis and fibrotic markers revealed that severe AKI-induced maladaptive tubular repair with immune cell infiltration and fibrosis. Tubular damage was restored completely in mild AKI rather than in severe AKI. Of note, persistent overexpression of NLRP3 was also found in severe AKI but not in mild AKI. In the severe AKI-induced chronic phase, there was a long-term high level of NLRP3 in serum or urine. Overt NLRP3 was mainly distributed in the abnormal tubules surrounded by inflammatory infiltrates and fibrosis, which indicated the maladaptive repair. Renal *Nlrp3* overexpression was correlated with infiltrating macrophages and fibrosis. Renal NLRP3 signaling-associated genes were upregulated after severe AKI by RNA-sequencing. Furthermore, NLRP3 was found increased in renal tubular epitheliums from CKD biopsies. Together, persistent NLRP3 overexpression was associated with chronic pathological changes following AKI, which might be a new biomarker for evaluating the possibility of AKI-CKD transition.

## Introduction

Acute kidney injury (AKI) is an increasingly common complication occurring in critically ill patients with high morbidity and mortality [1, 2]. Previous clinical studies demonstrate that AKI predisposes to the development and progression of chronic kidney disease (CKD), and the concept of AKI-CKD transition has been established [3–6]. Ischemia-reperfusion injury (IRI) is a common cause of clinically severe AKI [7–9]. Recently, continuing evidence supports that IRI-induced maladaptive tubular repair is the key that further contributes to permanent inflammation, progressive fibrosis, and eventually CKD [10–12]. In view of the refractory consequence of CKD, early detection and treatment are crucial for improving the prognosis of AKI and efficiently minimize AKI-CKD transition. However, how to early identify progressive AKI with tubular maladaptive repair is poorly understood. Renal biopsy, which is known as the clinical gold standard for diagnosing renal disease, cannot be widely performed in post-AKI patients without clinical indications [13]. Therefore, new noninvasive biomarkers are urgently needed to identify patients whether are at high risk for AKI-CKD transition or not.

Nod-like receptor protein 3 (NLRP3), an important member of the nucleotide-binding domain (NOD)-like receptor family, is a multi-protein complex and sensor in cells activated by damage-associated molecular patterns (DAMPs) [14]. Activated NLRP3 nucleates an inflammasome, leading to caspase 1-mediated proteolytic activation of the interleukin-1β (IL-1β) family of cytokines and induces inflammatory, pyroptotic cell death [15]. Of, note, a recent study indicated a possibility of the renal protective effect of NLRP3 knockout in IRI-induced AKI [16]. In addition, renal NLRP3 deficiency showed protective effects against kidney injury and played a similar effect as a reactive oxygen species (ROS) scavenger [17]. These findings indicate NLRP3 plays an unfavorable role in renal acute injury. However, whether NLRP3 is associated with AKI maladaptive repair has not been clarified yet. Here, we evaluated the expression of renal NLRP3 in the short- and long-term phases of the AKI mouse model, and investigated whether NLRP3 has a link to tubular maladaptive repair following AKI to become a new biomarker for early evaluating renal recovery and monitoring AKI-CKD transition.

## Results

### Severe ischemic AKI caused renal maladaptive repair with fibrosis

**Fig. 1:**
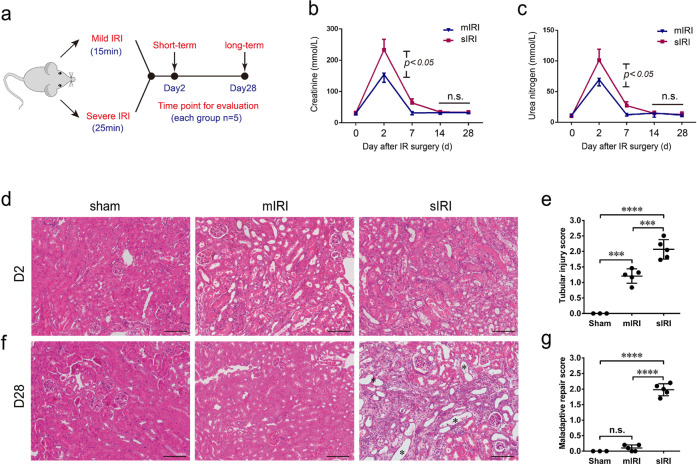
Short- and long-term outcomes after mild or severe IRI-induced AKI. **a** Schematic of the design of mild and severe AKI mice model. The mild and severe AKI were induced by 15 and 25 min of renal ischemic durations, respectively. Short- and long-term outcomes were evaluated in 2 and 28 days after surgery. **b** Serum creatinine and **c** blood urine nitrogen levels of two group mice subjected to mild and severe renal IRI at indicated time points. *n* = 5 for mild AKI and severe AKI mice, respectively. **d** Representative images of the renal cortex 2 days after surgery stained with HE. **e** Semi-quantitative scoring of renal tubular damage in kidneys at 48 h after surgery considering tubular dilation, brush border loss, tubular degeneration, tubular cast formation, and tubular necrosis. **f** Representative images of the renal cortex 28 days after surgery stained with HE. Asterisk represents the renal maladaptive-repaired tubules. **g** Semi-quantitative scoring of renal maladaptive repair in 28 days after surgery considering tubular atrophy, brush border loss, tubular degeneration, interstitial fibrosis. *n* = 3 mice for the sham group, *n* = 5 for mild and severe AKI per time-point group, respectively. Data are mean ± SD. ***P < 0.001, ****P < 0.0001. ns not significant. **d**, **f** Bar = 50 mm.

To investigate the long-term outcomes of AKI with different severity, an IRI-induced AKI mouse model was performed by using two different ischemia times (milder with 15 min and a longer 25 min) (Fig. 1a). In both mild and severe AKI mice, serum creatinine (Cr) and blood urea nitrogen (BUN) showed a notable increase within 48 h but subsequently declined to baseline during the next 7–14 days (Fig. 1b, c). Consistent with serum Cr and BUN, tubular damage was more severe in 25 min- versus 15 min-IRI mice (Fig. 1d, e). Besides, the renal tubular damage marker NGAL was significantly increased in severe IRI mice, compared to mild IRI mice (Supplementary Fig. S1a, b). Consistently, immunohistochemistry staining for NGAL also showed more expression in severe IRI group mice (Supplementary Fig. S1c). Although renal function restored basically, post-severe AKI mice exhibited structural defects in kidney tissue including atrophic tubules, and surrounded fibrosis on day 28 (Fig. 1f, g). On contrary, renal histology in post-mild AKI mice returned to normal in 28 days, suggesting a renal complete recovery (Fig. 1f, g). These results showed that severe AKI can cause incomplete recovery with fibrosis whereas serum Cr and BUN are not sensitive to early AKI-to-CKD transition.

### Sustained overexpression of renal NLRP3 following severe AKI

**Fig. 2:**
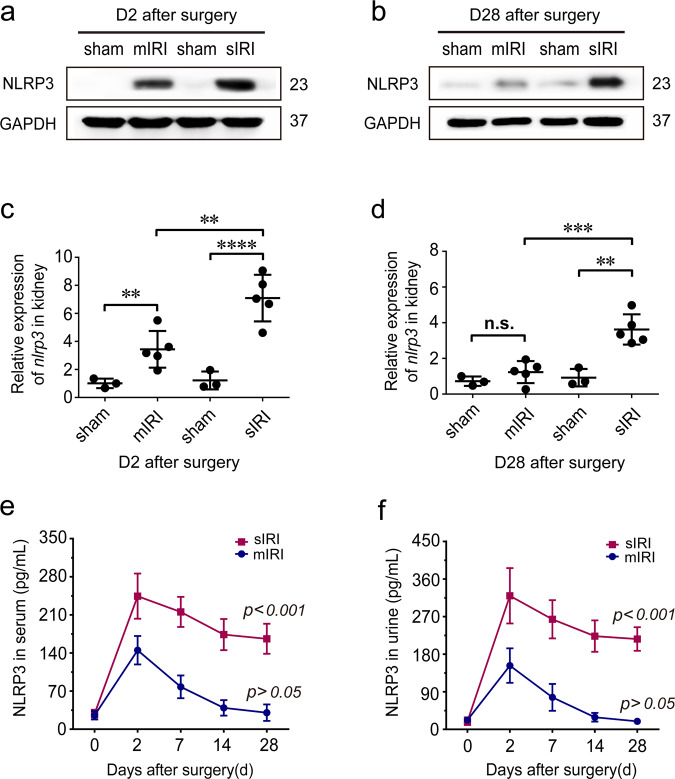
Dynamic expression of renal NLRP3 following AKI. **a**, **b** Representative western blot analysis of NLRP3 (23 kDa) protein in injured kidneys at indicated time points; GAPDH was used as a loading control. **c**, **d** Relative expression of NLRP3 mRNA in kidneys in injured kidneys at indicated time points. **e** Serum levels of NLRP3 from mild and severe group mice. ***P < 0.001 means significant versus baseline. **f** Urine levels of NLRP3 from mild and severe group mice. ***P < 0.001 means significant versus baseline. *n* = 3 mice for the sham group, *n* = 5 for mild and severe AKI per time-point group, respectively. Data are mean ± SD. **P < 0.01, ***P < 0.001, ****P < 0.0001. ns not significant.

Since the level of NLRP3 in long-term outcome after AKI is unknown, we determine the NLRP3 in serum, urine, and renal tissue following AKI. Western blotting showed renal NLRP3 increased in both mild and severe AKI mice after 2 days (Fig. 2a). However, in 28 days, renal NLRP3 was still overexpressed in severe AKI mice but not in the mild AKI group (Fig. 2b). As for mild AKI mice, renal NLRP3 expression decreased and returned to normal levels after 28 days (Fig. 2a, b). Consistently, renal expression of *Nlrp3* increased in both mild and severe AKI mice in early-stage after IRI. Whereas 28 days later, renal *Nlrp3* was still upregulated in severe AKI but not in mild AKI mice (Fig. 2c, d). Furthermore, similar results were also observed in serum or urine after AKI. Mild AKI mice presented a temporary increase of NLRP3 in serum or urine during the first week and subsequently returned to normal baseline, whereas severe AKI mice still kept high levels by the 28th day (Fig. 2e, f).

### NLRP3 overexpression was associated with maladaptive tubular repair and correlated with inflammatory infiltration and fibrosis

**Fig. 3:**
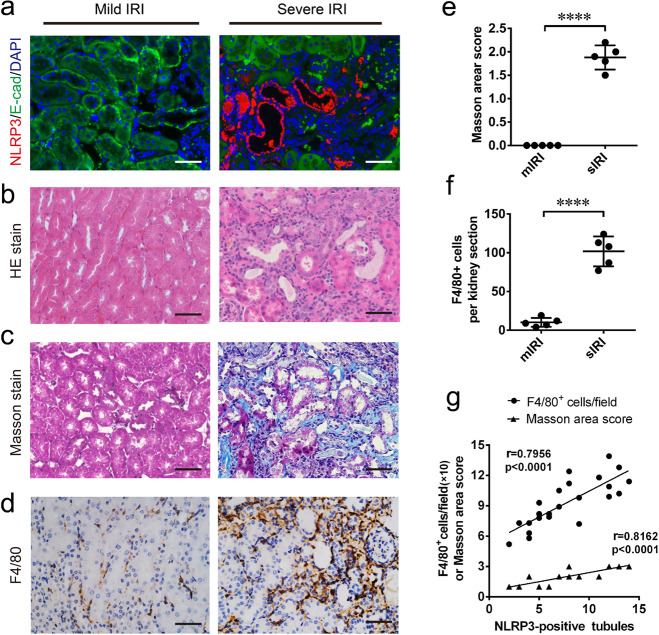
Tubular NLRP3 upregulation associated with the renal maladaptive repair, and correlated with inflammation and fibrosis. **a** Representative images of co-immunofluorescence staining of NLRP3 (red) and E-cadherin (green) in kidney section at chronic phase following AKI. **b** Representative images of cortex stained with HE. **c** Representative images of cortex stained with Masson trichrome. **d** Representative images of immunohistochemistry stain of F4/80 in kidney section. **e** Analysis and semi-quantification of Masson trichome evaluated by area scoring. **f** semi-quantification of F4/80-positive cells in per kidney section. **g** Correlation analysis of NLRP3-positive tubules with Masson trichome score (*r* = 0.8162) and F4/80-positive cells (*r* = 0.7956). 0.5 ≤ |r| < 0.8 defined as Moderately correlated. *n* = 3 mice for the sham group, *n* = 5 for mild and severe AKI per time-point group, respectively. Data are mean ± SD. ****P < 0.0001. **a**–**d** Bar = 20 mm.

To examine whether renal increased NLRP3 has a link to renal failed recovery, we examined the expressed location of NLRP3, as well as renal inflammatory infiltration and interstitial fibrosis by the 28th day after surgery. Double immunofluorescence staining showed that NLRP3 was mainly upregulated in abnormal tubular epithelial cells with negative cell-polarity-marker E-cadherin (Fig. 3a). The NLRP3-positive tubules were found with or without lotus tetragonolobus lectin (LTL) which is a proximal tubular marker (Supplementary Fig. S2). In combination with HE staining, these tubules were identified with maladaptive repair features, atrophy, dedifferentiation, surrounding by fibrosis, indicated tubular NLRP3 overexpression was associated with maladaptive repair (Fig. 3b). Notably, post-severe AKI mice showed maladaptive tubular repaired were surrounded by fibrosis lesions and F4/80-positive infiltrates, compared with mild AKI mice (Fig. 3c–f). Furthermore, NLRP3-positive tubules were correlated with renal fibrosis (*r* = 0.8162; *p* < 0.0001) and inflammatory infiltration (*r* = 07956; *p* < 0.0001), respectively (Fig. 3g, h). These data indicate that tubular NLRP3 overexpression was associated with the maladaptive repair, and also was correlated with renal inflammatory infiltration and fibrosis.

### Gene expression of renal NLRP3 signaling was upregulated following severe AKI

**Fig. 4:**
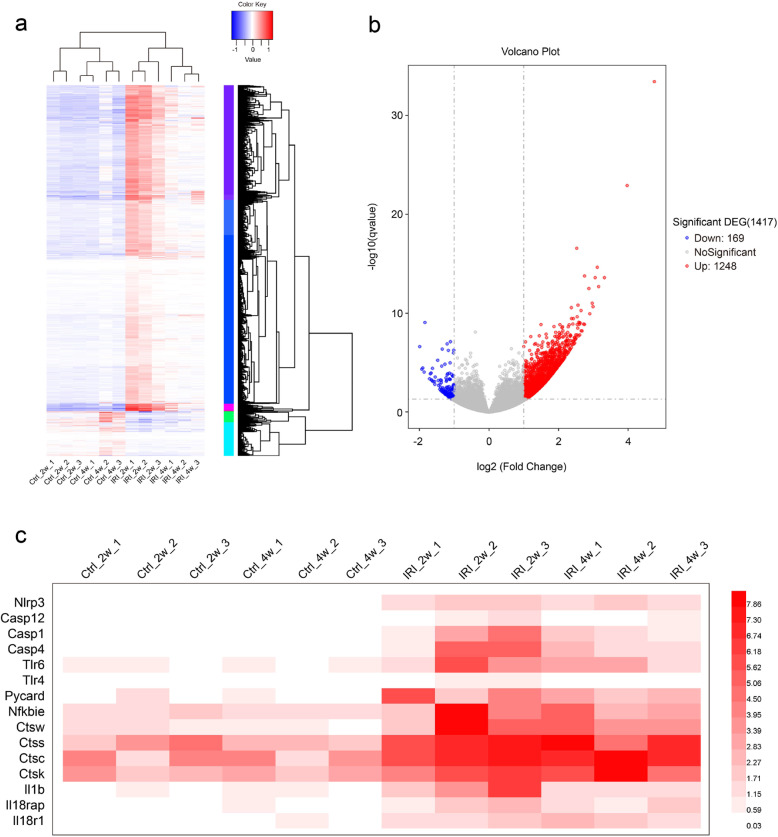
RNA-sequencing of renal NLRP3 signaling following AKI. **a** Heatmap of 1417 DEGs screened by limma package. Red areas represent highly expressed genes and blue areas represent lowly expressed genes in 2 or 4 weeks post-IRI group, compared with sham controls. **b** Volcano plot analysis identifies DEGs. Red dots represent upregulated genes and green dots represent downregulated genes in 2 or 4 weeks post-IRI group, compared with sham controls. **c** The expression of NLRP3 signaling-associated genes NLRP3 signaling-associated genes in sham control or IRI groups. DEG differentially expressed gene, IRI Ischemia/reperfusion injury.

NLRP3 inflammasome signaling activation is a key determinant of chronic inflammation in a variety of inflammatory cells and pathological conditions [18]. Here, we examined NLRP3 signaling in kidney tissues subjected to severe AKI by using RNA-sequencing. As shown in Fig. 4a, 2–4 weeks after IRI, 1417 DEGs in kidneys were screened in the heatmap by the limma package (adjusted *p* < 0.05, |logFC | ≥ 0.5). 169 genes were downregulated and 1248 genes were upregulated (Fig. 4b). Notably, NLRP3 signaling-associated genes in post-severe AKI mice were upregulated until 28 days (Fig. 4c).

### CKD patients represented high NLRP3 expression in renal biopsy specimens

**Fig. 5:**
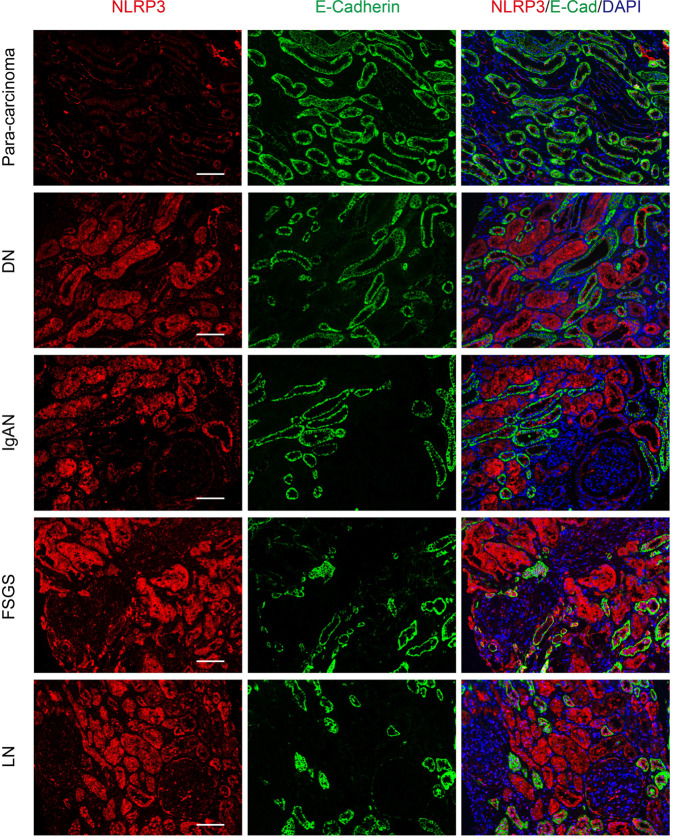
Immunofluoresence of NLRP3 in a renal biopsy from CKD-V patients. Double immunofluorescence staining with NLRP3 and E-cadherin was performed in CKD-V renal biopsies including diabetic nephropathy (DN), IgA nephropathy (IgAN), lupus nephritis (LN), and focal segmental glomerulosclerosis (FSGS). Para-carcinoma was as controls. Bar = 20 mm. CKD-V chronic kidney disease stage V.

Clinically, renal biopsy specimens of CKD patients who developed from previous AKI are rare. Given that, we evaluated NLRP3 expression in renal biopsy specimens from other common clinical CKD patients. These CKD patients were all developed chronic kidney disease, stage V (CKD-V) in the clinic. Double immunofluorescence staining also revealed that NLRP3 was markedly upregulated in E-cadherin-negative tubules from several CKD including diabetic nephropathy (DN), IgA nephropathy (IgAN), and lupus nephritis (LN) (Fig. 5). Although NLRP3 expression in E-cadherin-positive tubular epithelial cells from focal segmental glomerulosclerosis (FSGS) and LN was somewhat higher compared to DN, IgAN, we could find NLRP3 mainly expressed in E-cadherin-negative tubular epithelial cells (Fig. 5). These data suggest NLPR3 was elevated in common CKD renal biopsy specimens and it could be a marker for a chronic tubular lesion in CKD.

## Discussion

In our present study, we hypothesized that NLRP3 has a link to renal failed recovery after AKI. To test this hypothesis we applied the IRI mice model with different severity and explored the dynamic levels of NLRP3 in post-AKI mice. Here, we found sustained overexpression of NLRP3 in serum, urine, and kidney tissues for at least one month. Our results indicated tubular NLRP3 overexpression associated with maladaptive tubular repair, inflammation, and fibrosis. In addition, we also found that NLRP3 remarkably increased in renal biopsy specimens from common clinical CKD. Hence, our finding implies NLRP3 may contribute AKI progressive and can be used as a new biomarker to monitor AKI-CKD transition.

The NLRP3, as a cytoplasmic receptor, responds to an extensive array of molecules associated with cellular stress and associated with overproduction of proinflammatory cytokines [15]. Recent attention highlighted the protective role of NLRP3 deficiency in acute AKI outcomes [16, 19, 20]. However, it is less known whether NLRP3 play role in long-term AKI outcome. Our results revealed NLRP3 was upregulated in post-AKI kidneys for a long time, and its persistent overexpression was associated with the tubular maladaptive repair. An increasing number of clinical studies have indicated AKI may progress to CKD [21, 22]. Remarkably, tubular maladaptive repair has been considered as the driven factor exacerbating interstitial inflammation and fibrosis [6, 23–25]. The maladaptive tubular repair presented atrophic tubules, flattening epitheliums, loss of brush border, luminal dilatation. NLRP3 overexpression mainly occurred in these abnormal tubules with E-cadherin deficiency, suggesting epithelial polarity loss and epithelial dedifferentiation. Does the question arise which type of renal tubules exactly they are, proximal or distal? After double staining with NLRP3 and proximal tubular marker, we found NLRP3 was immunolabelled in both tubules with and without proximal tubular marker (Supplementary Fig. S2). It suggests that proximal tubules are involved in NLRP3 overexpression, but whether the other abnormal tubules without LTL are proximal tubules or not remains unclear because epithelial dedifferentiation also affects tubular characteristics to lose epithelial marker [26]. Since G2/M cell cycle arrest is considered an important player in maladaptive tubular repair after AKI, these epithelial cells are unable to proliferate and re-differentiate so that lose the normal function of conventional tubules, and become pathological tubules, which further promote kidney inflammation and fibrosis [27, 28]. In accordance with our findings, a recent study also highlighted that the typical NLRP3 promotes the pathophysiology of various kidney diseases by mediating inflammation, and this is likely a critical priming mechanism for renal fibrosis [29]. Hence, we thought NLRP3 was associated with progressive AKI and might promote renal chronic inflammation and fibrosis.

**Fig. 6:**
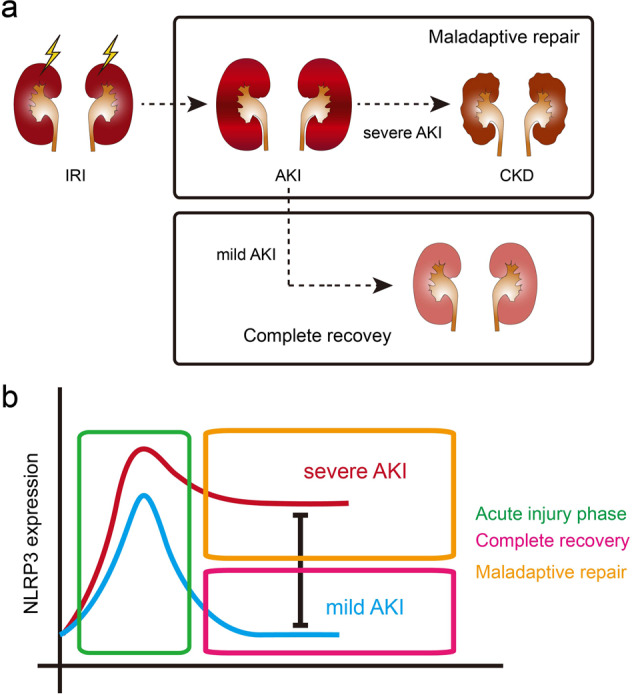
A summary of NLRP3 dynamic alteration in initial tissue injury and subsequent repair of the kidney after acute kidney injury. **a** Renal maladaptive and incomplete repair would occur when kidney subjected to severe IRI. **b** Renal maladaptive and incomplete repair would be accompanied with higher expression levels of NLRP3 in AKI-to-CKD progression.

It is known the functional loss in AKI patients can be transient [21, 28]. In the present study, both mild and severe AKI mice showed renal function restored completely in 4 weeks after I/R. However, when AKI enters the chronic process with renal maladaptive repair, persistent pathological characterization is, in turn, accelerating kidney loss of function eventually [30–32]. Hence, serum Cr and BUN are not sensitive to early chronic progression after AKI. We hypothesized NLRP3 can be an indicator for early evaluating AKI recovery and monitoring long-term outcomes like KIM-1 and NGAL [9, 33]. Renal RNA-sequencing showed that expression of NLRP3 signaling genes in post-AKI kidneys were all upregulated, compared to control groups. As previous studies reported, once renal maladaptive repair occurs after AKI, the abnormal tubules can be existence and interstitial inflammation, fibrosis will be even exacerbated progressively [3, 4, 27]. Therefore, a high level of NLRP3 in vivo may imply incomplete repair after AKI and further predict a high risk for chronic progression (Fig. 6). In addition, the pathological study from AKI to CKD is still poorly understood, partly because renal biopsies are rarely performed in patients who are clinically improving. Although we could not provide the evidence about NLRP3 in clinical biopsy specimens from AKI-progressed CKD, we also found NLRP3 increasing associated with renal tubular lesions by immunofluorescence of other CKD biopsies e.g., DN, IgAN, LN, and FSGS. AKI and its progression are especially prominent in renal tubular lesions [4, 27]. Thus, high levels of NLRP3 after AKI can be considered as a new biomarker for chronic tubular lesions predicting AKI-CKD transition. Our results may have clinical implications for the prognostic assessment of humans with AKI.

Taken together, sustained overexpression of NLRP3 can be an indicator of the poor prognosis of murine AKI. However, this requires confirmation in further clinical studies. Furthermore, as a biomarker, we hope to provide a better understanding of the reasonable dynamic range of NLRP3 in AKI patients, it needs to be studied in depth in the future.

## Materials and methods

### Animals

Male 8-week-old C57BL/6J mice were obtained from the Shanghai SLAC Laboratory Animal Center. The mice were housed in the *Animal Facility of Shanghai General Hospital* under a 12-h light/dark pattern with free access to food and water. The animal study was approved by the Ethics Committee of Shanghai General Hospital. All experiments were performed in accordance with ARRIVE guidelines [34].

### IR-induced AKI models

Ischemic AKI was induced in male mice of 12-week old as described recently [23]. Briefly, mice were anesthetized by intraperitoneal injection of sodium pentobarbital at a dose of 40 mg/kg. Preemptive analgesia with buprenorphine (0.2 mg/100 g) was used [35]. Body temperature was maintained at 37 °C and monitored during surgery using a temperature controller with a heating pad (TCAT-2, YUYAN Instruments, Shanghai, China). Next, through a midline incision, mild or severe AKI was induced after right unilateral nephrectomy by clipping the pedicles of the remaining left kidney with a non-traumatic aneurysm clip (Rivard Life Science Co. Ltd., Shenzhen, China) for 15 or 25 min, respectively. These two periods of ischemia were based on other earlier IRI studies with mice [9, 36–38]. Then, the clip was released and reperfusion was verified visually. After surgery mice had free access to water and chow. Perioperative hydration by applying body-warm sterile physiological saline solution and analgesia by subcutaneous buprenorphine (0.1 mg/kg) was provided for each mouse. Two or twenty-eight days after reperfusion, different time-point group mice (*n* = 5 per IRI group) were sacrificed by overdose of sodium pentobarbital and additionally cervical dislocation, and kidney and blood samples were collected for further analysis, respectively. Sham control mice underwent the same operation by exposing unilateral renal artery without ischemia (*n* = 3 per sham group). The left kidneys were immediately quickly removed and processed for histological evaluation, protein and RNA extraction. The kidneys were divided into three portions. The upper pole of the kidney tissue was frozen and used for western blotting. The middle part of the kidney was immersed in 4% phosphate-buffered saline (PBS)–buffered formalin for histology, and the other left tissue was snap-frozen in liquid nitrogen for RNA preparation.

### Histological assessments

To assess renal histology, all kidney samples were immersed and fixed overnight in 4% paraformaldehyde and then dehydrated through an ethanol series, and paraffin-embedded. Renal sections (2 μm) were stained with hematoxylin and eosin (HE) and Masson’s trichrome stain. Histomorphometric analysis was performed to semi-quantitively evaluate the score of acute tubular injury and chronic fibrosis. Slides were examined under a light microscope (×400 magnification). The examination was performed to 10 randomly selected views of each slide by a local nephropathologist, who was blinded to the experimental assignments. The pathological parameters of acute tubular injury by HE staining include: loss of brush border, luminal dilatation, epithelial simplification, urinary cylinder/cast, epithelial regeneration changes, detachment of epithelium, and fracture of the tubular basement membrane, tubular karyolysis, and interstitial infiltration. Similarly, the parameters of renal maladaptive repair include: tubule atrophy, interstitial fibrosis, inflammatory cell infiltration. Each histological parameter was graded from 0 to 3 according to the distribution of lesions: 0 = none; 1 = <25%; 2 = 25–50%; 3 = >50% [39]. Next, the average values of all parameters in the 10 views of each kidney slice were counted for analysis and comparison as Supplementary Table S1.

### Deidentified human kidney specimens

All sections of human kidney biopsy specimens used in the study were obtained with approval from the Department of Nephrology of Shanghai General Hospital (Shanghai, China). All patients provided written informed consent. Patients who suffered from CKD-V were recruited to examine NLRP3 expression in their biopsy samples. Biopsy specimens of controls (para-carcinoma) and twelve patients with CKD (seven men and five women; ages 35–66 years old; the serum creatinine ranged from 786 to 1180 µmol/L) including three cases of DN, three cases of IgAN, three cases of LN, and three cases of FSGS were fixed in paraffin and sectioned at 5 µm. The sections were analyzed by immunofluorescence with antibodies against NLRP3 and E-cadherin for further analysis.

### Immunohistochemistry and immunofluorescence

Paraformaldehyde-fixed, paraffin-embedded 2-µm-thick sections were deparaffinized and rehydrated. After antigen retrieval, sections were incubated overnight with antibodies against F4/80 (1: 200, CST, USA) at 4 °C. followed by biotinylated secondary antibody (Dako, Carpinteria, CA, United States) for 60 min at 37 °C. Then, 3, 3-diaminobenzidine was used as the chromogen. Finally, the slides were counterstained with hematoxylin and mounted after dehydration. F4/80-positive cells were quantified by counting the number of stained cells per high-power field (HPF) (×400) with 10 captured images included in each slide. The antibody of neutrophil gelatinase-associated lipocalin (NGAL) (1:500; Abcam, UK) was used to evaluate AKI severity.

The following primary antibodies for immunofluorescence were used: anti-NLRP3 (1:100; Abcam, UK); anti-E-cadherin (1:100; CST, USA). All incubations were performed in a humid chamber. For fluorescence visualization of bound primary antibodies, sections were further incubated with appropriate Cy3-conjugated or Alexa 488-conjugated secondary antibodies (1:500; Jackson ImmunoResearch Laboratories) for 1 h in a humid chamber at room temperature. Immunofluorescence imaging was performed using Zeiss 510 NLO Meta.

### Renal function and NLRP3 measurement

Renal function was assessed by using the kits of creatinine and blood urine nitrogen (JIANCHENG Bioengineering Institute, Nanjing, China). The serum and urine levels of NLRP3 from AKI mouse model or clinic CKD patients were determined by using the mouse or human NLRP3 ELISA kits respectively, according to the manufacturer’s protocols. All test indicators were performed in triplicate.

### Western blotting

Kidney tissues were crushed using a Precellys 24 homogenizer (Peqlab, Erlangen, Germany) and were lysed with RIPA buffer (Sigma-Aldrich, St. Louis, MO) supplemented with protease inhibitor and phosphatase inhibitor cocktail (Thermo Scientific, Massachusetts, USA) at 4 °C for 30 min. Twenty-five microgram protein samples were separated by 10% SDS-PAGE. After semi-dry transfer, the membranes were blocked with 5% nonfat milk and incubated with primary antibody overnight at 4 °C. Then, the membranes were probed with HRP-conjugated secondary antibody for 30 min at room temperature. The protein bands were detected with ECL reagent (NcmECL Ultra, NCM Biotech). Relative band densities of the proteins were analyzed by using ImageJ software (NIH). The primary antibodies used include: anti-NLRP3 (1:800; Abcam, UK), anti-NGAL (1:1000; Abcam, UK), GAPDH were used as internal controls of renal tissues.

### Quantitative real-time PCR

Quantitative Real-Time PCR (qRT-PCR) was performed as described earlier [40]. Briefly, total RNA was isolated from kidney cortex tissue using an RNeasy RNA isolation kit (Qiagen). The quality and concentration of renal tissue RNA were determined by a NanoDrop-1000 spectrophotometer (Thermo Fisher Scientific). Two micrograms of total renal tissue RNA were transcribed to cDNA (Applied Biosystems). Then, quantitative analysis of target mRNA expression was calculated using the relative standard curve method. SYBR green analysis was conducted using an Applied Biosystems 7500 Sequence Detector (Applied Biosystems). The expression levels were normalized to GAPDH. Primer sequences are provided as follows:

**Table:** 

	Forward	Reverse
m*Nlrp3*	5-AGAAGAGACCACGGCAGAAG-3	5-CCTTGGACCAGGTTCAGTGT-3
m*gapdh*	5-GATGTCATCATACTTGGCAGGTTT-3	5-GCATGGCCTTCCGTGTTC-3

### RNA-sequencing

We performed RNA-sequencing as described previously [41]. Total RNA was isolated from renal tissue as described in the *Method* of *qRT-PCR*. The concentration, quality, and integrity of RNA were checked by using Nanodrop and Bioanalyzer (RNA 6000 Nano Kit, Agilent Technologies, Santa Clara, CA). Five micrograms of high-quality tissue RNA were used to obtain poly A–enriched RNA by using the NEXTflex Poly(A) Kit (Bioo Scientific, Austin, TX). Next, the mRNA concentration was measured by using Qubit (HS RNA assay kit, Agilent Technologies), and 50 ng was used to prepare mRNA libraries by using the NEXTflex Rapid Directional mRNA-Seq Kit (Bioo Scientific). The library concentration and size were measured by using the KAPA Library Quantification Kit (Kapa Biosystem, Wilmington, MA) and Bioanalyzer, respectively. The libraries were then subjected to 100 bp paired-end next-generation sequencing (Illumina, SanDiego, CA). To visualize the distribution of reads on the NLRP3 signaling genes, BedGraph files were generated from RNA-sequencing data by using HemI 1.0-Illustrator software (The CUCKOO Workgroup, School of Life Science and Technology, Huazhong University of Science and Technology, China).

### Statistical analysis

Statistical analyses were performed using GraphPad Prism 6.0 (GraphPad Software). All data are presented as mean ± SD and *p*-values of <0.05 were considered as statistically significant. The *p*-values in the figures are denoted as follows: ns *p* > 0.05, *p < 0.05, **p < 0.01, ***p < 0.001 and ****p < 0.0001. Two-group comparisons were performed using the Student’s *t*-test, and multiple groups were evaluated by one-way ANOVA multiple comparisons post hoc test.

## Supplementary information

Supplementary Figure S1

Supplementary Figure S2

Supplementary figure legends

Supplementary Table S1

Supplementary table legend

Author Contribution statement

## Data Availability

All data generated or analyzed during this study are included in this published article and its supplementary information files.
