# Supplementary figures and images for: NLRP3 associated with chronic kidney disease progression after ischemia/reperfusion-induced acute kidney injury
Source: Cell Death Discov. 2021 Oct 29;7:324. doi: 10.1038/s41420-021-00719-2 (PMC8556399; doi:10.1038/s41420-021-00719-2)

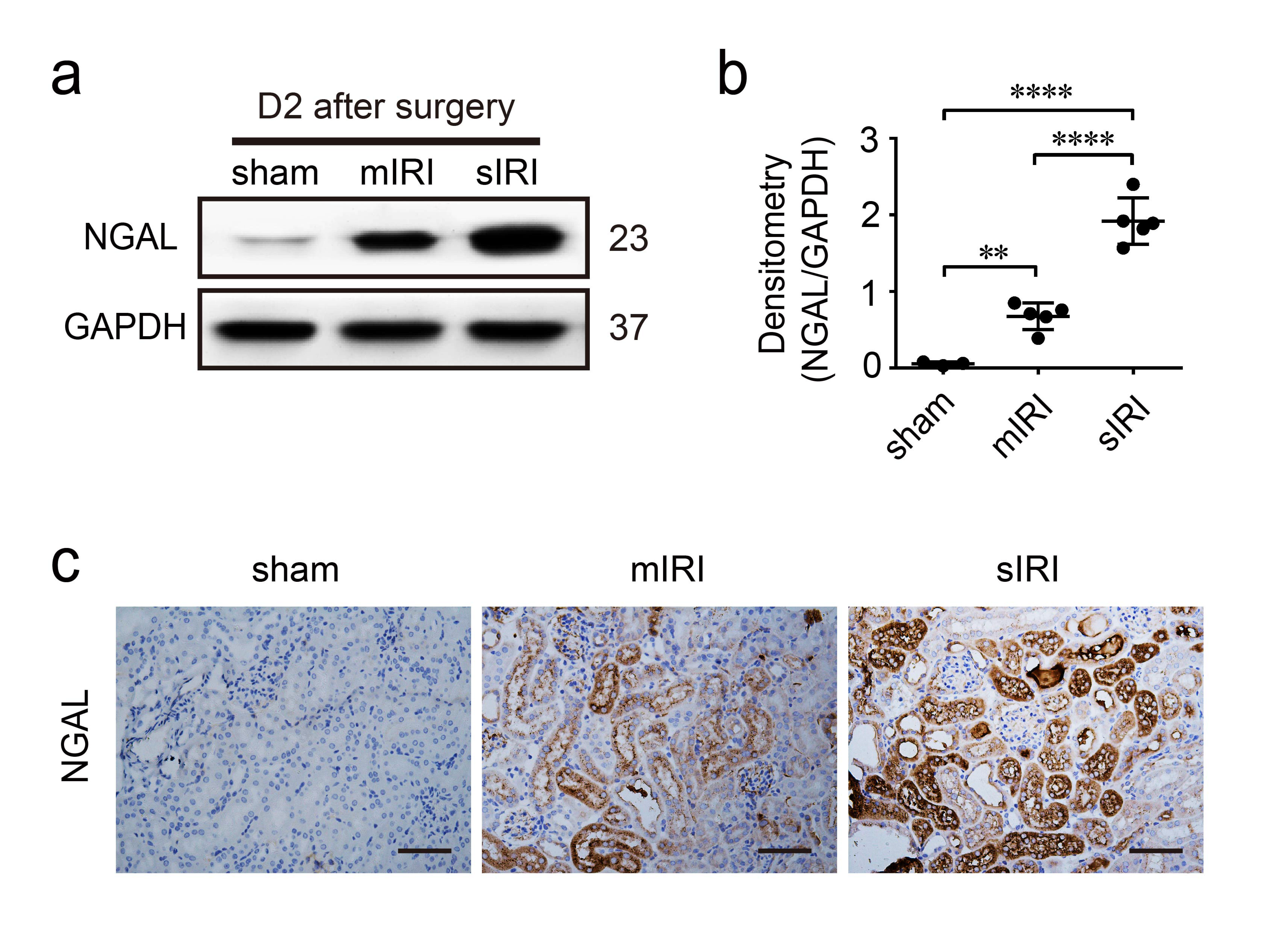

Supplement: Supplementary file 1 — Supplementary Figure S1 [file 41420_2021_719_MOESM1_ESM.tif]

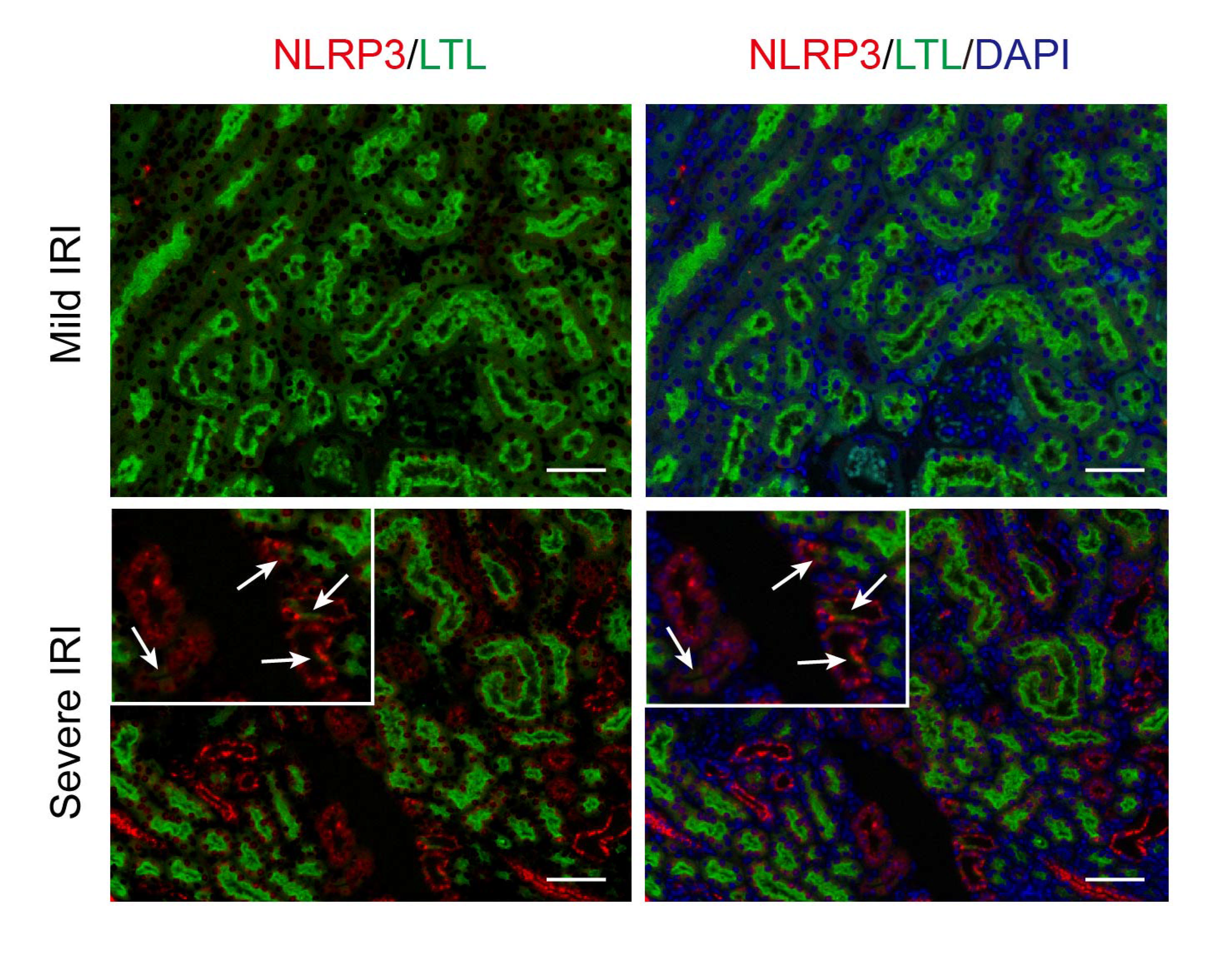

Supplement: Supplementary file 2 — Supplementary Figure S2 [file 41420_2021_719_MOESM2_ESM.tif]

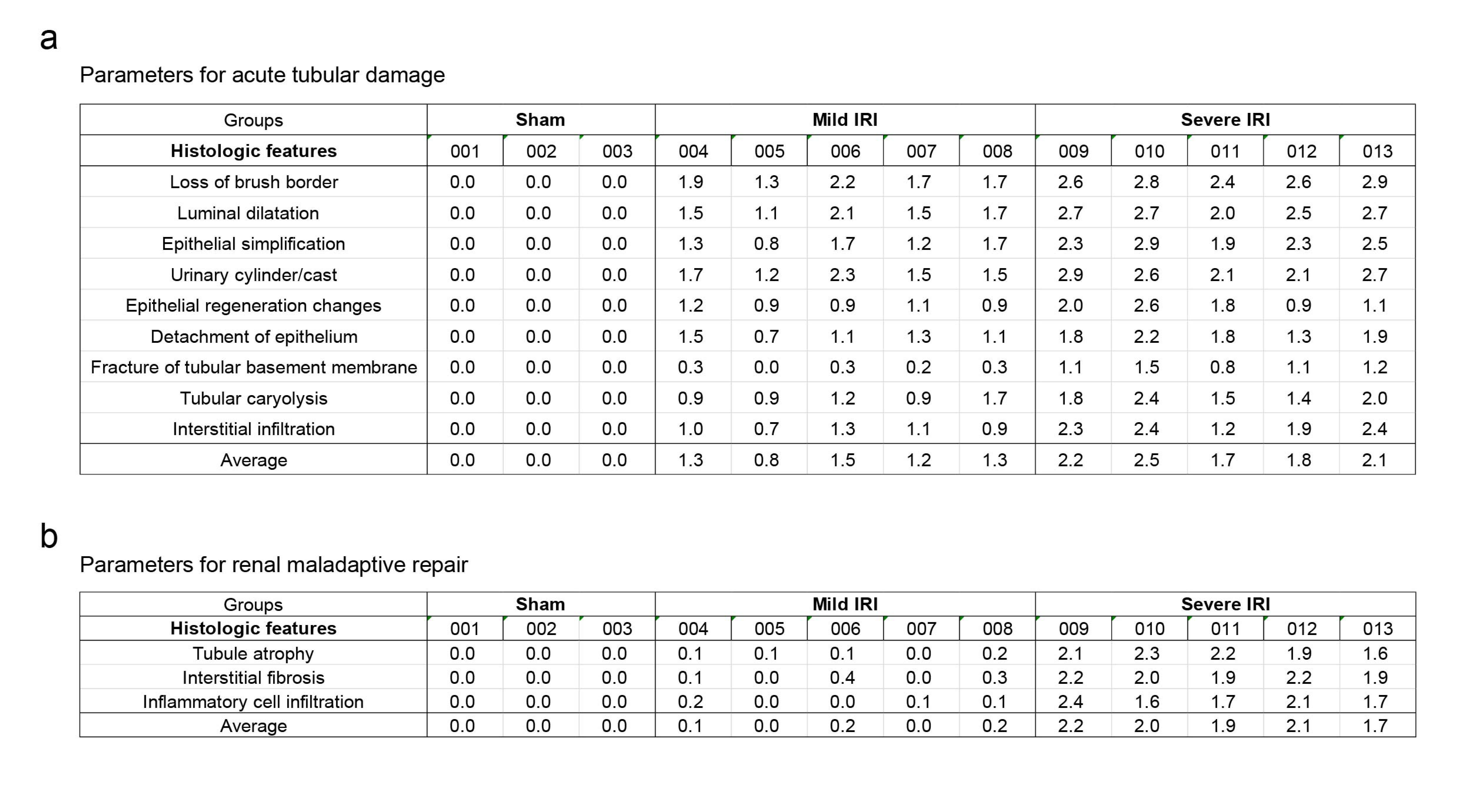

Supplement: Supplementary file 4 — Supplementary Table S1 [file 41420_2021_719_MOESM4_ESM.tif]
