# Supplementary material for: NLRP3 associated with chronic kidney disease progression after ischemia/reperfusion-induced acute kidney injury
Source: Cell Death Discov. 2021 Oct 29;7:324. doi: 10.1038/s41420-021-00719-2 (PMC8556399; doi:10.1038/s41420-021-00719-2)
Supplement: Supplementary file 3 — Supplementary figure legends [file 41420_2021_719_MOESM3_ESM.pdf]

Supplementary figure legends

Supplementary Figure S1. Renal NGAL expression in mild or severe AKI mice.

(a) Western blot examined the change of NGAL in murine kidney tissue with mild or severe IRI. (b) Representative images of NGAL staining in injured kidneys. Scale bar=20 μm in all images. All values are means ± SD. $^{**}p < 0.01$ and $^{****}p < 0.0001$ defined as significant.

Supplementary Figure S2. Kidney section stained with NLRP3 and LTL.

Twenty-eight days after IRI or sham surgery, murine kidneys were stained with NLRP3 and lotus tetragonolobus lectin (LTL) which is a proximal tubular marker. Scale bar=20 μm in all images.
