# Supplementary material for: NLRP3 associated with chronic kidney disease progression after ischemia/reperfusion-induced acute kidney injury
Source: Cell Death Discov. 2021 Oct 29;7:324. doi: 10.1038/s41420-021-00719-2 (PMC8556399; doi:10.1038/s41420-021-00719-2)
Supplement: Supplementary file 5 — Supplementary table legend [file 41420_2021_719_MOESM5_ESM.pdf]

Supplementary table legends

Supplementary Table S1. Evaluation of histopathological parameters to renal damage and maladaptive repair.

The pathological parameters of (a) acute tubular injury and (b) renal maladaptive repair were set in the table. The each histological parameter was graded from 0 to 3 according to the distribution of lesions: 0=none; 1=<25%; 2=25–50%; 3=>50%. The average values of all parameters in the 10 random views of each kidney slice was counted for analysis and comparison.
