# Supplementary material for: NLRP3 associated with chronic kidney disease progression after ischemia/reperfusion-induced acute kidney injury
Source: Cell Death Discov. 2021 Oct 29;7:324. doi: 10.1038/s41420-021-00719-2 (PMC8556399; doi:10.1038/s41420-021-00719-2)
Supplement: Supplementary file 6 — Author Contribution statement [file 41420_2021_719_MOESM6_ESM.pdf]

DECLARATION OF CONTRIBUTIONS TO ARTICLE

ADMC

Manuscript Number:	Journal Name:	
CDDISCOVERY-21-2131R1	*Cell Death Discovery*	(the 'Journal')

Proposed Title of the Contribution:

NLRP3 associated with chronic kidney disease progression after ischemia/reperfusion-induced acute kidney injury (the 'Contribution)

Author(s):

Zhihuang Zheng, Kexin Xu, Chuanlei Li, Chenyang Qi, Yili Fang, Nan Zhu, Jinfang Bao, Zhonghua Zhao, Qing Yu, Huijuan Wu and Jun Liu (the 'Authors')

For all *CDDis*covery articles, each person named as an author in the published version must be able to show he or she has contributed substantially to the article.

Authorship credit should be based on 1) substantial contributions to conception and design, acquisition of data, or analysis and interpretation of data; 2) drafting the article or revising it critically for important intellectual content; and 3) final approval of the version to be published. Authors should meet conditions 1, 2 and 3.

Any person who cannot be shown to have made a substantial contribution to the article cannot be listed as an author in the final version. The name of any person who is deemed to have made a minor contribution can, however, appear in the Acknowledgments section of the article.

Please complete the table below to indicate the contributions of all named authors to the manuscript.

Author Full Name:	Specification of Contribution to the Manuscript:
Zhihuang Zheng	performed animal models, and analyzed data and drafted the manuscript.
Kexin Xu	performed renal HE staining, immunohistochemistry, immunofluorescence.
Chuanlei Li	western blotting, qPCRs, and measured the levels of serum Cr and BUN
Chenyang Qi	performed renal RNA-sequencing and analysis
Yili Fang	Histopathological evaluation
Nan Zhu	collected cinical renal biopsy specimens
Jinfang Bao	supervised the all data analysis
Zhonghua Zhao	performed pathological techniques.
Qing Yu	Helped design the experiments
Huijuan Wu	supervised the experimental work and revised the manuscript
Jun Liu	designed experiments and helped write, revised the manuscript

Please complete the table below to indicate the contributions of all named authors to the figures.

Figure 1:

Zhihuang Zheng performed AKI models, did statistical analysis and prepared panel a, e, g. Kexin Xu and Yili Fang generated the histology data and prepared panel d, f. Zhonghua Zhao supply the pathological technology. Chuanlei Li measured serum Cr and BUN levels and prepared panel b, c. Jun Liu, Huijuan Wu, and Qing Yu supervised the experimental work.

Figure 2:

Chuanlei Li performed western blotting, qPCRs, and prepared panel a-d. Zhihuang generated the ELISA data and prepared panel e, f. Jun Liu, Huijuan Wu, and Qing Yu supervised the experimental work.

Figure 3:

Kexin Xu performed histology, immunohistochemistry, and prepared panel a-d. Zhihuang Zheng did statistical analysis and prepared panel e, f, g. Zhonghua Zhao supply the pathological technology. Jun Liu, Huijuan Wu, and Qing Yu supervised the experimental work.

Figure 4:

Chenyang Qi performed the RNA-seq experiments and prepared panel a, b, c. Huijuan Wu, supervised her work.

Figure 5:

Nan Zhu collected cinical renal biopsy specimens. Zhihuang Zheng and Kexin Xu performed immunofluorescence. Jinfang Bao supervised the experiments work.

Figure 6:

Zhihuang Zheng draft the pattern diagram and did the work for Supplementary Figure S1-2. Yili Fang did the histological evaluation as shown in Supplementary Table S1

Signed for and on behalf of the Author(s):	Print Name:	Date:
Jun Liu	Jun Liu	27.09.2021
